# Gender, Sexual Orientation, and Intersectionality in Oral Health Care

**DOI:** 10.1177/00220345251392145

**Published:** 2025-12-19

**Authors:** G.H. Soares, C.L. Randall, D. Haag, B. Poirier, G.G. Nascimento, L. Jamieson

**Affiliations:** 1 Australian Research Centre for Population Oral Health, Adelaide Dental School, The University of Adelaide, Australia; 2 University of Washington School of Dentistry, University of Washington, Seattle, WA, USA; 3 National Dental Research Institute Singapore, National Dental Centre Singapore, Singapore, Singapore

**Keywords:** sexual and gender minorities, gender identity, equity, healthcare disparities, health inequities, intersectional framework

## Abstract

This study uses an intersectional framework to examine how race, gender, and sexual orientation jointly influence access to dental care in the United States. Leveraging cross-sectional data from the All of Us Research Program, we applied multilevel analysis of individual heterogeneity and discriminatory accuracy to assess disparities in dental service use and affordability across 30 intersectional strata. Results showed substantial inequities, with racialized, gender-diverse, and sexual minority individuals facing greater barriers to care. While most disparities were explained by additive effects, the findings highlight the importance of intersectionality in revealing how layered disadvantage shapes oral health outcomes. This study contributes to a more nuanced understanding of oral health equity by integrating gender and sexual orientation into intersectional health research.

## Introduction

Individuals are born into preexisting social structures that shape cultural norms, define acceptable behaviors, and construct the meanings attached to experiences and identities (Nedel and Bastos 2020). These structures (eg, race, gender, sexuality, class) do not operate independently; they are mutually constitutive. For instance, race is experienced through the intersecting lenses of gender, sexuality, and class, just as gender is shaped by sexuality, class, and race (Bowleg et al 2025). These interdependent structures create complex and oppressive social hierarchies, determining not only who holds power but also who is systematically marginalized. Disparities in access to critical resources such as education, employment, housing, and health care are produced, sustained, and normalized through structural discriminatory processes that establish compounding cycles of disadvantage (Williams et al 2019; Beech et al 2021).

As oral health disparities remain a global challenge that appears intractable (Wen et al 2022), the field of oral health research has increasingly acknowledged the need to interrogate the systems of power that give rise to structural inequalities (Bastos et al 2020; Lala et al 2021; Fleming et al 2023). Intersectionality provides a framework to critically examine how different social structures and positions intersect to produce compound disadvantage and shape oral health outcomes (Muirhead et al 2020; Bastos et al 2022; Madera et al 2023; Soares et al 2025). Emerging as a lens by which to illuminate the experiences of women of color (Crenshaw 1991), intersectionality rapidly evolved into a robust framework for understanding the layered nature of discriminatory structures for groups experiencing multiple forms of marginalization (Collins 2015). Intersectionality challenges the notion that marginalization operates along a single axis and emphasizes the interconnected nature of these social structures and categories (Jordan-Zachery 2007). From an intersectional perspective, race, gender, and sexuality cannot be analytically separated without losing complexity. As a theoretical framework and analytical tool, intersectionality recognizes that oppression and privilege converge to produce unique outcomes that are both context dependent and embedded within broader structural hierarchies. Intersectionality offers not only a foundation for advancing equity but also a politics of survival that is centered on the liberation of marginalized groups (Hancock 2007).

While socioeconomic and racial disparities in oral health have been widely documented (Bastos et al 2023), previous studies have largely overlooked the joint influence of race, gender, and sexual orientation on oral health. In this article, we aim to demonstrate the relevance of incorporating categories of gender, sexual orientation, and race within an intersectionality framework to investigate inequities in access to dental care.

## Methods

We leveraged data from the All of Us Research Program, a large-scale research initiative launched in 2015 in the United States, to center gender and sexuality as key analytical social constructs in an intersectional analysis of oral health inequities. Anchored in the principles of diversity, inclusion, and equitable outcomes, All of Us offers an unprecedented opportunity to investigate health disparities through an intersectional lens. The initiative aims to generate data from more than 1 million individuals, with a strong emphasis on oversampling groups historically underrepresented in health research (more than 75% of participants identify with marginalized communities based on race, ethnicity, sex, gender, sexual orientation, age, income, disability, access to care, geography, or educational attainment) (All of Us Research Program Investigators et al 2019; Mapes et al 2020; Bianchi et al 2024). As of 29 May 2025 (Controlled Tier, version 6), All of Us included 627,584 total participants. Information on health care utilization was available for 305,857 participants (Appendix Fig S1).

## Outcomes

We examined dental service utilization and ability to afford dental care as independent outcomes. Dental service utilization was measured by asking participants whether they have seen or talked to a dentist or orthodontist about their own health in the past 12 mo. The ability to afford dental care was assessed by asking participants whether, at any point in the past 12 mo, they needed dental care (including checkups) but were unable to afford it. Response options for each question included “yes,” “no,” and “don’t know.” Participants who answered “don’t know” were excluded.

## Intersectional Categories

We constructed 30 strata representing unique intersections of race, gender, and sexual orientation. Race was categorized as Black, Hispanic, multiracial, other (aggregated due to small sample sizes: Asian, American Indian and Alaska Native, Native Hawaiian and Other Pacific Islander, and Middle Eastern and North African), and White. To accurately construct categories of gender, we combined information on sex assigned at birth and gender identity. Gender was classified as cisgender man, cisgender woman, or gender diverse (including trans, nonbinary, and other gender identities). Sexual orientation was classified as heterosexual or sexual minority (including lesbian, gay, and bisexual). Information on race, gender identity, and sexual orientation was self-reported.

## Analysis

We followed standard recommendations for conducting intersectional multilevel analysis of individual heterogeneity and discriminatory accuracy (MAIHDA) (Evans et al. 2024). The multilevel approach in MAIHDA allows the analysis of intersectional social positions as contextual strata, rather than as isolated individual attributes. When modeling binary outcomes, MAIHDA involves fitting a series of 2-level logistic regressions with individuals at level 1 nested within social strata at level 2. The first model is an empty multilevel model including random intercepts for the social strata. The empty multilevel model is used to partition the variance found in the outcome into the variance between strata (social intersections) and the variance within strata (individuals). The variance partition coefficient (VPC) is a key statistic derived from multilevel models that quantifies the proportion of the total individual variance that lies between strata. The VPC is calculated as the variance at the strata level divided by the total variance. In binary models, the residual variance at the individual level is fixed (assumed to be π^2^/3 ≈ 3.29 on the logistic scale). VPC values range from 0 to 1 and are often expressed as a percentage. High values indicate that individuals within the same stratum tend to have very similar probabilities of experiencing the outcome, while differing substantially from individuals from other strata. Conversely, a VPC value of zero implies that strata are not useful for explaining differences in the probability of experiencing the outcome.

The second MAIHDA model included fixed main effects for race, gender, and sexual orientation. No interaction terms were included. This model provides information on how much of the between-strata variance is explained by the main effects of individual variables, while the random intercept for strata at level 2 captures the remaining unexplained variance between-stratum after accounting for the main effects. This approach aims to quantify the extent to which observed inequities between social intersections are described by additive versus multiplicative effects. All models were adjusted by age.

While the exponentiated coefficients expressed as odds ratios (ORs) in the second model reflect the contribution of each variable while holding the others constant, our primary interest was in interpreting the VPC. In this model, the VPC represents the proportion of total variance attributable to strata (interaction effects) after accounting for the additive main effects of race, gender, and sexual orientation. We also calculated the proportional change in variance (PCV) to quantify the extent to which the between-strata variances decreased between the empty model and the second model by adjusting for the additive effects. A PCV value less than 100% indicates that interaction effects are necessary for accurately capturing observed inequities between strata. We predicted the strata-level probability of experiencing the outcome based on the intersectional strata to describe and illustrate disparities across intersectional groups. Analyses were conducted in RStudio. This study was approved by the All of Us Research Program’s institutional review board. The reporting of this study conforms to the STROBE statement.

## Results

The analytical sample included 228,886 participants with complete information on intersecting categories and dental service utilization (model A) and 264,244 participants with complete information on intersecting categories and ability to afford dental care (model B). Table 1 displays the distribution of participants across the adopted social categories and the corresponding outcome prevalence. Overall, 73.1% of participants reported dental service utilization in the previous 12 mo, whereas 82.4% reported being able to afford dental care. White, cisgender, and heterosexual participants consistently reported higher utilization and affordability of dental care compared with racialized, gender-diverse, and sexual minority groups.

**Table 1.** Distribution of the Analytical Sample according to Main Social Positions.

	Dental Care Utilization			Able to Afford Dental Care		
	Overall Sample		Yes	Overall Sample		Yes
	*n*	%	%	*n*	%	%
Total	228,886	100	73.1	264,244	100	81.9
Race						
White	159,764	69.8	78.3	182,601	69.1	84.9
Black	20,284	8.9	57.1	23,865	9.0	75.7
Hispanic	22,760	9.9	56.3	27,214	10.3	77.1
Other	9,871	4.3	71.8	11,810	4.5	84.1
Multiracial	16,207	7.1	66.4	18,754	7.1	73.1
Gender						
Cis men	75,409	32.9	73.4	89,656	33.9	86.4
Cis women	150,980	66.0	73.1	171,808	65.0	80.4
Gender diverse	2,497	1.1	59.2	2,780	1.1	68.8
Sexual orientation						
Heterosexual	205,414	89.7	73.8	237,866	90.0	83.4
Sexual minorities	23,472	10.3	67.1	26,378	10.0	73.2

We examined the number of participants in each of the 30 strata representing unique intersections of race, gender, and sexual orientation. Stratum sizes ranged from 26 to 93,588 participants (median 923) for dental service utilization and from 32 to 105,694 participants (median 1,077) for the ability to afford dental care. For both outcomes, 29 of the 30 strata included more than 50 participants (Appendix Table S1). Sensitivity analysis accounting for missing responses showed that even under extreme scenarios (±10 percentage points), overall patterns of dental service utilization and affordability remained similar (Appendix Tables S2–S3). These findings suggest that the observed disparities are robust and unlikely to be substantially affected by selection bias.

The empty MAIHDA models revealed substantial clustering at the stratum level, with VPC values of 14.3% for dental service utilization and 11.7% for the ability to afford dental care. The additive main effects for dental service utilization (model 1) and ability to afford dental care (model 2) are presented in Table 2. Gender-diverse participants had 28% lower odds of utilizing dental services and 39% lower odds of being able to afford dental care compared with cis men, holding all other variables constant. Sexual minority individuals had 32% lower odds of being able to afford dental care compared with heterosexual participants. Cis women had 27% lower odds of being able to afford dental care compared with cis men. While interpreting these model estimates provides insights into additive patterns (that is, the independent contribution of each variable to the outcome), it may obscure critical findings that emerge only when examining intersections.

**Table 2.** Parameter Estimates for Utilization of Dental Service (Model A) and Ability to Afford Dental Care (Model B).

	Model A		Model B	
	OR	95% CI	OR	95% CI
Intercept	1.34	1.14–1.57	2.77	2.51–3.05
Race				
White	1		1	
Black	0.44	0.36–0.53	0.65	0.58–0.73
Hispanic	0.51	0.42–0.61	0.75	0.67–0.84
Other	0.93	0.76–1.14	1.17	1.04–1.32
Multiracial	0.71	0.59–0.85	0.65	0.58–0.72
Gender				
Cis men	1		1	
Cis women	1.05	0.91–1.20	0.73	0.68–0.79
Gender diverse	0.72	0.60–0.86	0.61	0.54–0.69
Sexual orientation				
Heterosexual	1		1	
Sexual minority	1.08	0.95–1.23	0.74	0.68–0.80
VPC (empty model)	14.3		11.7	
VPC	2.3		0.6	
PCV	83.9		95.0	

CI, confidence interval; OR, odds ratio; PCV, proportional change in variance; VPC, variance partition coefficient.

The VPC in both additive models decreased substantially compared with the empty model. The proportion of the total variance between strata that accounted for additive main effects is expressed by PCV values of 83.7% (model 1) and 95.0% (model 2). The remaining variance between strata is attributable to interaction effects.

We examined the predicted probabilities of dental service utilization and affordability across intersecting categories (Appendix Fig S1–S2). The top 5 strata for dental service use included White cisgender heterosexual women (60.8%), White cisgender heterosexual men (57.2%), other-race cisgender heterosexual women (59.4%), White cisgender sexual minority men (57.2%), and other-race cisgender sexual minority men (55.6%). In contrast, the 5 strata with the lowest predicted probabilities included Black gender-diverse sexual minority participants (38.2%), Hispanic gender-diverse sexual minority participants (35.6%), Black gender-diverse heterosexual individuals (34.5%), Black cisgender heterosexual men (31.1%), and Hispanic gender-diverse heterosexual individuals (30.4%).

The 5 strata with the highest predicted probabilities of being able to afford dental care were observed among White cisgender heterosexual men (76.3%), other-race cisgender heterosexual men (73.9%), other-race cisgender heterosexual women (72.1%), other-race cisgender sexual minority men (70.1%), and White cisgender heterosexual women (68.5%). The 5 strata with the lowest predicted probabilities included multiracial gender-diverse heterosexual individuals (51.5%), Hispanic gender-diverse sexual minority individuals (50.5%), multiracial gender-diverse sexual minority participants (49.6%), Black cisgender sexual minority women (48.9%), and multiracial, cisgender sexual minority women (47.3%).

## Discussion

This study found substantial inequities in the utilization and affordability of dental care across intersectional strata defined by race, gender, and sexual orientation. In the simple MAIHDA model, the social strata explained a substantial proportion of the total variance in outcomes between individuals. Findings not only demonstrate that structural systems of power such as colonialism, racism, patriarchy, cisnormativity, and heteronormativity are central to understanding how oral health inequities are produced but also emphasize that these systems and their effects on health cannot be fully understood in isolation.

In both study models, the between-stratum variances were largely explained by the contribution of additive independent effects of race, gender, and sexual orientation rather than by their interaction effects. These findings suggest that inequities between strata are mostly driven by the cumulative layering of multiple axes of advantage and/or disadvantage. It should be noted that the absence of substantial multiplicative (intersectional) effects does not imply that intersectionality is not present. While MAIHDA offers a powerful approach for scaling up quantitative intersectional analysis, it cannot fully account for the embodied, relational, and contextual dimensions of interlocking systems of power. Quantitative intersectional models must be embedded within a critical epistemological framework that is attentive to the social structures that shape how inequities are produced and experienced (Bowleg 2012).

In addition to revealing broader patterns of disadvantage, our findings provide nuanced insights into how different forms of advantage and disadvantage intersect to shape access to resources. White cisgender men who identify with a minority sexual orientation had one of the highest probabilities of utilizing dental services across all strata, comparable to heterosexual White cisgender men and women. In contrast, Black cisgender heterosexual men had one of the lowest probabilities. While privilege associated with Whiteness seems to mitigate some of the disadvantages related to gender and sexual minority status, structural barriers rooted in racism continue to disproportionately limit access to dental care for Black people.

The consistent pattern of racial inequities across strata underscores structural racism as a key determinant of disparities in access to health care (Bastos et al 2023; Bastos 2025). Gender and sexual orientation also play significant roles, but their effects are not uniform or independent. Rather, they interact with racialized structures in complex ways. Gender-diverse and sexual minority individuals often experience barriers to care related to stigma, discrimination, or institutional erasure from policies and systems (Raisin et al 2023; Burchell et al 2024). The magnitude and nature of these barriers are shaped by race and other social positions. This means that the disadvantage faced by a gender-diverse person of color may be compounded by racism and socioeconomic marginalization, resulting in experiences that are both qualitatively and quantitatively distinct from those of their White counterparts.

Findings can guide interventions to reduce oral health inequities, with particular focus on groups experiencing the greatest disadvantage (particularly racialized gender-diverse and racialized sexual minority groups). Policies should promote affirmative and inclusive dental care that is culturally safe for all. Efforts should also address systemic discrimination, including racism, sexism, transphobia, and homophobia, rather than focusing solely on high-risk groups. Importantly, structural reforms should be assessed for their impact on reducing intersectional inequities in oral health (Anticona et al 2024).

This study represents an important shift in oral health research, moving away from a normative binary definition of gender. To the best of our knowledge, this is the first study to articulate how race, gender, and sexuality affect oral health outcomes. Another strength of this study is the use of a large dataset, which allows for the stratification of individuals across a wide range of categories. Several important limitations should be noted. We were unable to include intersex individuals or disaggregate all racial and gender identities due to small sample sizes. Most strata were sufficiently large to obtain reliable estimates. For the single stratum with fewer than 50 participants, MAIHDA provides conservative estimates that tend toward the overall fixed-effect mean. This property, known as shrinkage, reduces the likelihood of overestimating intersectional effects (Evans et al 2024). In addition, the analysis was limited to participants with complete data across all social categories and outcomes. Further, because data are from the United States only, generalizability to other populations may be limited. However, the intersectional framework used is broadly applicable and can guide similar research globally, helping to identify groups experiencing compounded disadvantage and inform context-specific interventions.

While socioeconomic status is an established driver of access to dental care, the primary aim of our study was to examine the intersectional effects of race, gender, and sexual orientation, addressing an important gap in the dental literature. Our decision to focus on the intersection of race, gender, and sexual orientation was driven by both theoretical and empirical considerations. Race, gender, and sexual orientation act as broader determinants that not only shape access to dental care but also account for potential differences across mediators such as socioeconomic status. Including socioeconomic status as a social category would drastically increase the number of strata, leading to smaller sizes and unreliable estimates. While one might speculate whether the lack of adjustment for variables such as income and education constitutes a limitation, we conceptualized these factors as mediators through which structural forces related to race, gender, and sexual orientation affect access to dental care. Adjusting for these factors could result in overadjustment, potentially obscuring existing intersectional inequities. Finally, race, gender, and sexuality represent important domains of identity that are relatively stable throughout the lifespan. By focusing on these social constructs, our study contributes to revealing inequities in oral health care that extend beyond socioeconomic factors.

## Conclusion

This study emphasizes the relevance of integrating gender and sexual orientation into intersectional oral health research. In convergence with race and other structural axes of oppression, broader and more inclusive definitions of gender and sexuality provide a renewed perspective to explore the complexities of how inequities in oral health are produced and sustained. Intersectionality challenges single-axis models of analysis that treat social locations as separate variables and shifts the focus toward the central issue of addressing the root causes of inequity.

## Author Contributions

G.H. Soares, contributed to conception and design, data analysis and interpretation, drafted and critically revised manuscript; C.L. Randall, D. Haag, B. Poirier, G.G. Nascimento, L. Jamieson, contributed to conception and design, data interpretation, critically revised manuscript. All authors gave final approval and agree to be accountable for all aspects of the work.

## Supplemental Material

sj-docx-1-adr-10.1177_00220345251392145 – Supplemental material for Gender, Sexual Orientation, and Intersectionality in Oral Health Care Supplemental material, sj-docx-1-adr-10.1177_00220345251392145 for Gender, Sexual Orientation, and Intersectionality in Oral Health Care by G.H. Soares, C.L. Randall, D. Haag, B. Poirier, G.G. Nascimento and L. Jamieson in Advances in Dental Research
