# Supplementary material for: Gender, Sexual Orientation, and Intersectionality in Oral Health Care
Source: Adv Dent Res. 2025 Dec 19;33(1):17–21. doi: 10.1177/00220345251392145 (PMC12717284; doi:10.1177/00220345251392145)
Supplement: sj-docx-1-adr-10.1177_00220345251392145 – Supplemental material for Gender, Sexual Orientation, and Intersectionality in Oral Health Care [file sj-docx-1-adr-10.1177_00220345251392145.docx]

**Supplementary files**

**Gender, sexual orientation and intersectionality in oral healthcare**

**The compounding effects of race, gender, and sexuality on access to oral healthcare**

Racial inequities in oral health are well documented, with racism contributing to increased barriers to accessing services, lower-quality care, and historical marginalisation within healthcare systems (Jamieson et al., 2021). Structural racism contributes to these disparities by limiting access to dental insurance, concentrating providers in affluent and predominantly White areas, and perpetuating culturally unsafe care (Jamieson et al., 2021; Smith et al., 2021). Evidence from Australia indicates that individuals who experienced racism are approximately 90% more likely to report barriers to accessing healthcare (Bastos et al., 2018). In the United States, women from racialised backgrounds are up to four times more likely to report never having visited a dental clinic compared to White women (Gupta et al., 2019).

Sexual orientation and gender identity further affect experiences of care, as LGBTQIA+ individuals frequently face stigma and discrimination while accessing dental services (Tharp et al., 2022). Findings from a large national survey of transgender and gender diverse individuals in the Unites States show that one-third of participants who saw a healthcare provider had at least one negative experience related to their gender identity, ranging from being refused treatment to being verbally, physically or sexually abused (James et al., 2016). Nearly one-quarter of participants avoided healthcare due to anticipated discrimination (James et al., 2016). Negative experiences with health care providers are substantially higher for trans people who identify as American Indian, Middle Eastern or multiracial compared to their White and Asian counterparts (James et al., 2016). Trans people of colour also experience reduced access to dental services, highlighting how intersecting systems of racism and transphobia can shape and exacerbate oral health disparities (Clermont et al., 2025).

All of Us Study: Participant enrolment began in May 2018 and includes individuals aged 18 years or older recruited through a network of more than 340 sites across the United States (All of Us Research Program Investigators et al. 2018). The data repository includes electronic health records, health surveys, genomic data, anthropometric measurements, personal digital information, and collection and analysis of biospecimens.

Figure S1. Flowchart of participant inclusion for analysis of utilisation of dental services and affordability of dental care.

All of Us Study (n=627,584)

Analysed (n= 228,886)

Valid answers (n=244,513)

Excluded:

♦ Missing sexual orientation (n=9,401)

♦ Missing race (n=5,014)

♦ Missing gender (n=2,321)

Utilisation of dental services

(n=288,181)

Excluded:

♦ Skipped (n=42,577)

♦ Don’t know (n=1,091)

Healthcare utilisation survey (n=305,857)

Analysed (n=264,244)

Valid answers (n=282,471)

Excluded:

♦ Missing sexual orientation (n=10,762)

♦ Missing race (n=6,015)

♦ Missing gender (n=2,795)

Affordability of dental care

(n=305,857)

Excluded:

♦ Skipped (n=21,481)

♦ Don’t know (n=1,905)

Table S1. Number of participants across strata for each outcome.

| Strata | Race | Gender | Sexual Orientation | Utilisation | Affordability |
| --- | --- | --- | --- | --- | --- |
| 1 | White | Cis men | Heterosexual | 49,434 | 58,300 |
| 2 | White | Cis men | Sexual minority | 5,683 | 6,394 |
| 3 | White | Cis women | Heterosexual | 93,588 | 105,694 |
| 4 | White | Cis women | Sexual minority | 9,357 | 10,346 |
| 5 | White | Gender diverse | Heterosexual | 310 | 347 |
| 6 | White | Gender diverse | Sexual minority | 1,392 | 1,520 |
| 7 | Black | Cis men | Heterosexual | 4,891 | 6,009 |
| 8 | Black | Cis men | Sexual minority | 581 | 678 |
| 9 | Black | Cis women | Heterosexual | 13,779 | 15,974 |
| 10 | Black | Cis women | Sexual minority | 906 | 1,061 |
| 11 | Black | Gender diverse | Heterosexual | 59 | 71 |
| 12 | Black | Gender diverse | Sexual minority | 68 | 72 |
| 13 | Hispanic | Cis men | Heterosexual | 5,570 | 7,050 |
| 14 | Hispanic | Cis men | Sexual minority | 795 | 937 |
| 15 | Hispanic | Cis women | Heterosexual | 15,255 | 17,896 |
| 16 | Hispanic | Cis women | Sexual minority | 941 | 1,094 |
| 17 | Hispanic | Gender diverse | Heterosexual | 100 | 125 |
| 18 | Hispanic | Gender diverse | Sexual minority | 99 | 112 |
| 19 | Other | Cis men | Heterosexual | 3,400 | 4,244 |
| 20 | Other | Cis men | Sexual minority | 390 | 458 |
| 21 | Other | Cis women | Heterosexual | 5,555 | 6,491 |
| 22 | Other | Cis women | Sexual minority | 416 | 487 |
| 23 | Other | Gender diverse | Heterosexual | 26 | 32 |
| 24 | Other | Gender diverse | Sexual minority | 84 | 98 |
| 25 | Multiracial | Cis men | Heterosexual | 3,921 | 4,726 |
| 26 | Multiracial | Cis men | Sexual minority | 744 | 860 |
| 27 | Multiracial | Cis women | Heterosexual | 9,465 | 10,838 |
| 28 | Multiracial | Cis women | Sexual minority | 1,718 | 1,927 |
| 29 | Multiracial | Gender diverse | Heterosexual | 61 | 69 |
| 30 | Multiracial | Gender diverse | Sexual minority | 298 | 334 |

Figure S2. Strata-level predicted prevalence of dental service utilisation.


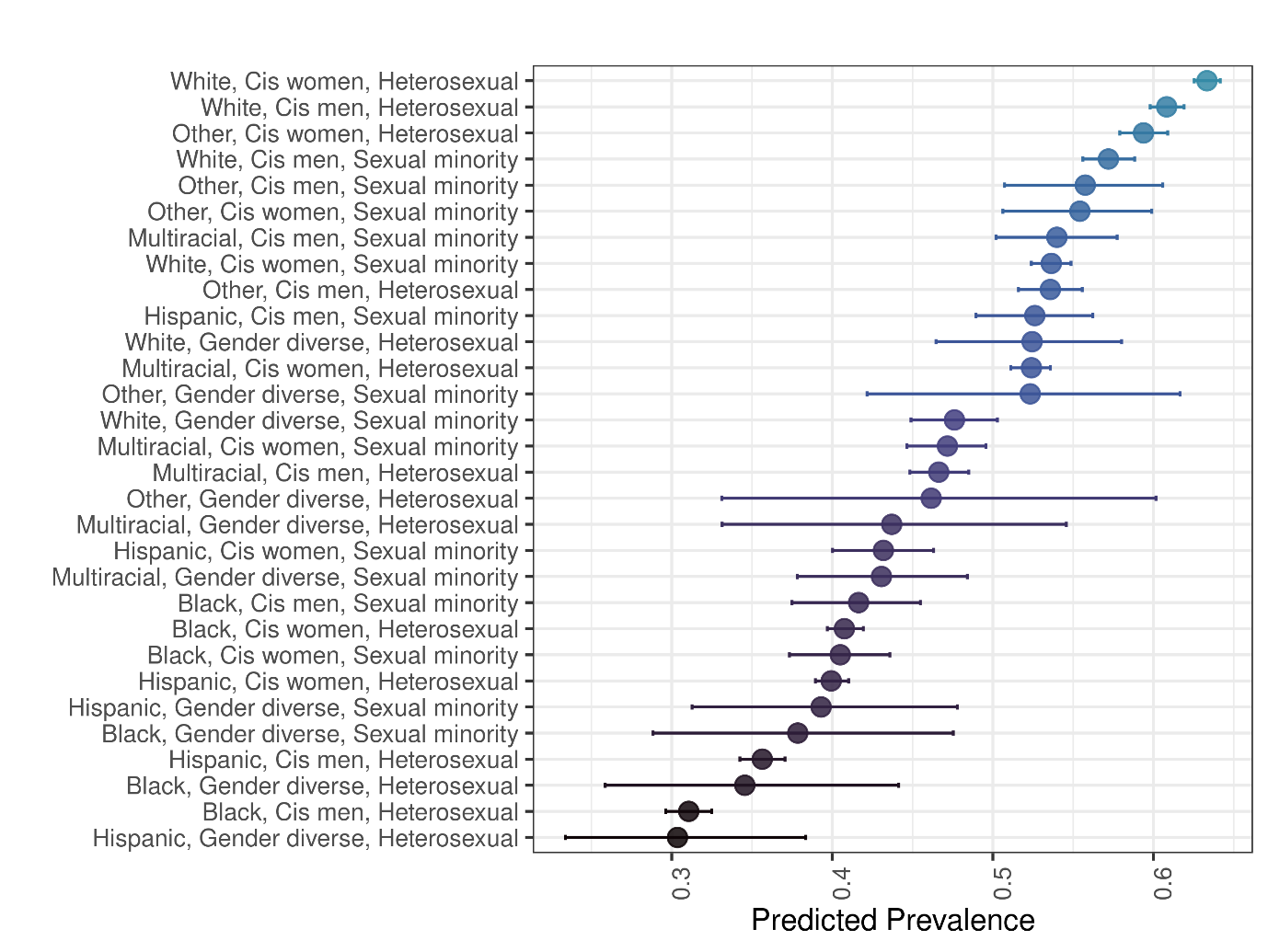


Figure S3. Strata-level predicted prevalence of being able to afford dental care.


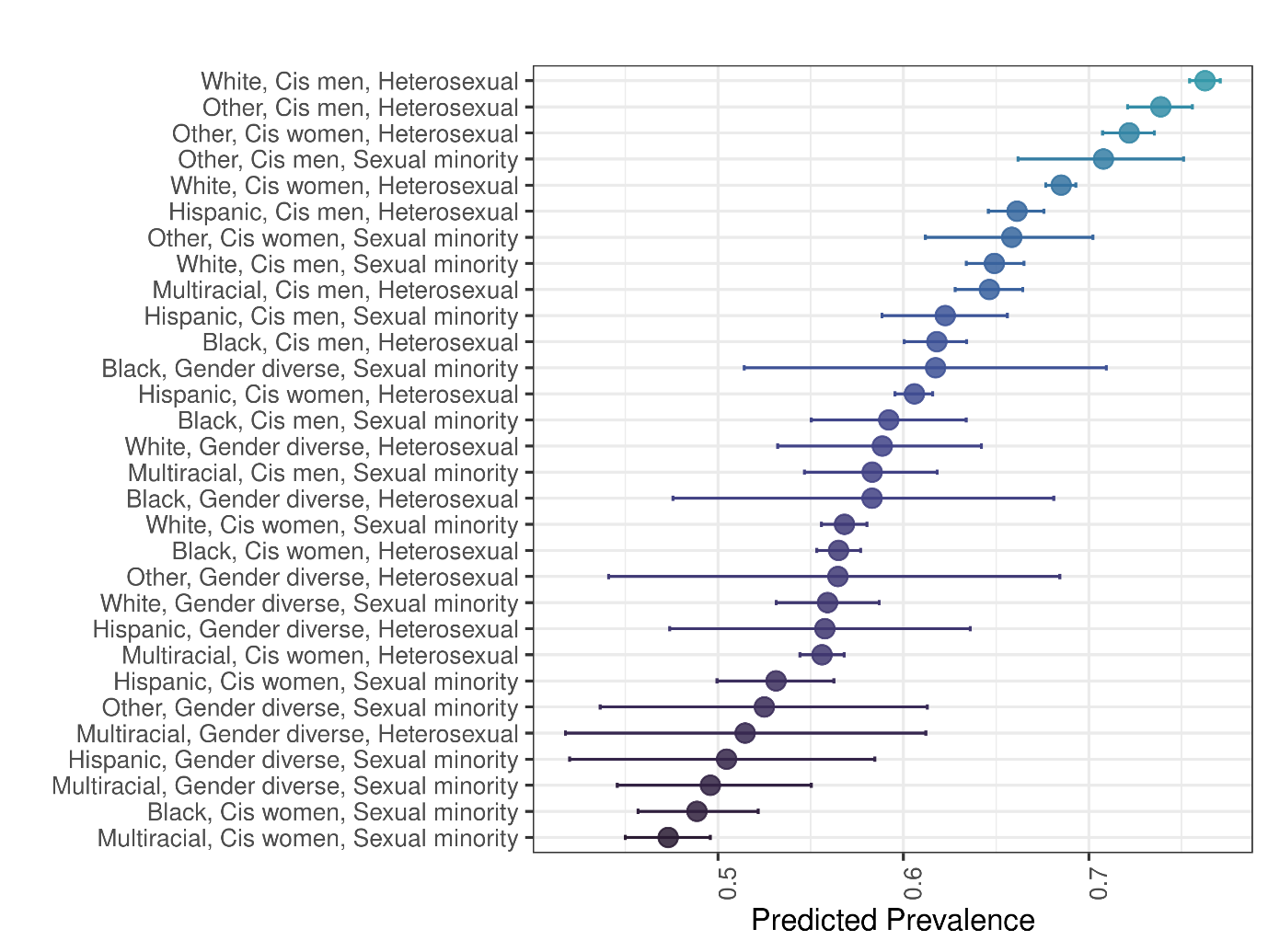


Table S2. Quantitative bias analysis for utilisation of dental care under different missing scenarios.

|  | % Missing | Observed prevalence | Scenario: Missing -10pp | Scenario:  Missing +10pp |
| --- | --- | --- | --- | --- |
| Race |  |  |  |  |
| White | 14.7 | 78.3 | 76.8 | 79.8 |
| Black | 19.3 | 57.1 | 55.2 | 59 |
| Hispanic | 15.2 | 56.3 | 54.8 | 57.8 |
| Other | 13.4 | 71.8 | 70.5 | 73.1 |
| Multiracial | 11.5 | 66.4 | 65.2 | 67.6 |
| Gender |  |  |  |  |
| Cis men | 17.4 | 73.4 | 71.7 | 75.1 |
| Cis women | 14.0 | 73.1 | 71.7 | 74.5 |
| Gender diverse | 7.1 | 59.2 | 58.5 | 59.9 |
| Sexual orientation |  |  |  |  |
| Heterosexual | 15.8 | 73.8 | 72.2 | 75.4 |
| Sexual diverse | 9.2 | 67.1 | 66.2 | 68 |

Table S3. Quantitative bias analysis for affordability of dental care under different missing scenarios.

|  | % Missing | Observed prevalence | Scenario: Missing -10pp | Scenario:  Missing +10pp |
| --- | --- | --- | --- | --- |
| Race |  |  |  |  |
| White | 7.3 | 84.9 | 84.2 | 85.6 |
| Black | 9.4 | 75.7 | 74.8 | 76.6 |
| Hispanic | 8.1 | 77.1 | 76.3 | 77.9 |
| Other | 6.8 | 84.1 | 83.4 | 84.8 |
| Multiracial | 5.9 | 73.1 | 72.5 | 73.7 |
| Gender |  |  |  |  |
| Cis men | 8.2 | 86.4 | 85.6 | 87.2 |
| Cis women | 7.3 | 80.4 | 79.7 | 81.1 |
| Gender diverse | 4.6 | 68.8 | 68.3 | 69.3 |
| Sexual orientation |  |  |  |  |
| Heterosexual | 7.8 | 83.4 | 82.6 | 84.2 |
| Sexual diverse | 5.3 | 73.2 | 72.7 | 73.7 |

**References**

Bastos JL, Harnois CE, Paradies YC. Health care barriers, racism, and intersectionality in Australia. Soc Sci Med. 2018 Feb;199:209-218.

Clermont D, Nieto V, Alpert E, Yao E, Cothron A. How socioeconomic and structural barriers influence dental care among transgender people. J Public Health Dent. 2025 Mar;85(1):73-83. doi: 10.1111/jphd.12655.

Gupta A, Feldman S, Perkins RB, Stokes A, Sankar V, Villa A. Predictors of dental care use, unmet dental care need, and barriers to unmet need among women: results from NHANES, 2011 to 2016. J Public Health Dent. 2019 Dec;79(4):324-333.

James SE, Herman JL, Rankin S, Keisling M, Mottet L, Anafi M. The Report of the 2015 U.S. Transgender Survey. Washington, DC: National Center for Transgender Equality; 2016.

Jamieson L, Peres MA, Guarnizo-Herreño CC, Bastos JL. Racism and oral health inequities; An overview. EClinicalMedicine. 2021 Apr 6;34:100827.

Smith PD, Wright W, Hill B. Structural Racism and Oral Health Inequities of Black vs. non-Hispanic White Adults in the U.S. J Health Care Poor Underserved. 2021;32(1):50-63. doi: 10.1353/hpu.2021.0007. PMID: 33678680.

Tharp G, Wohlford M, Shukla A. Reviewing challenges in access to oral health services among the LGBTQ+ community in Indiana and Michigan: A cross-sectional, exploratory study. PLoS One. 2022 Feb 25;17(2):e0264271. doi: 10.1371/journal.pone.0264271. PMID: 35213637; PMCID: PMC8880834.
